# Turkish reliability and validity of Neurorehabilitation Experience Questionnaire

**DOI:** 10.1590/1806-9282.20241781

**Published:** 2025-06-02

**Authors:** Abdurrahim Yıldız, Esen Cicekli

**Affiliations:** 1 Sakarya University of Applied Sciences, Faculty of Health Sciences, Department of Physiotherapy and Rehabilitation – Turkey.; 2 Akyazı State Hospital, Department of Neurology – Turkey.

**Keywords:** Neurological rehabilitation, Reliability, Satisfaction, Validity

## Abstract

**OBJECTIVE:**

The aim of this study was to evaluate the Turkish validity and reliability of the Neurorehabilitation Experience Questionnaire, which assesses the experiences of patients receiving neurorehabilitation.

**METHODS:**

Neurological patients receiving rehabilitation between October 2023 and August 2024 were included in the study. Reliability was assessed by calculating Cronbach's alpha for internal consistency and using the test–retest method to measure stability. Validity was assessed through construct and criterion validity with convergent validity tested with the Patient Care and Rehabilitation Perception Scale in Elderly Patients.

**RESULTS:**

The mean age of the participants was 54.88 years and BMI was 28.55. The test–retest reliability of the Neurorehabilitation Experience Questionnaire showed a strong positive correlation (r=0.932, p<0.001), demonstrating the stability of the scale. A moderate, significant correlation was found between Neurorehabilitation Experience Questionnaire and Perception of Patient Care and Rehabilitation in Older Patients Scale scores (r=0.440, p<0.001). Moreover, both the first and second assessments of the Neurorehabilitation Experience Questionnaire were positively correlated with the corresponding Perception of Patient Care and Rehabilitation in Older Patients Scale assessments, strengthening the convergent validity of the questionnaire.

**CONCLUSION:**

The Turkish version of the Neurorehabilitation Experience Questionnaire is a reliable and valid instrument for measuring neurorehabilitation experience in Turkish-speaking patients. The application of this questionnaire in clinical settings may improve patient-centered care by facilitating a better understanding of patient experiences and satisfaction in neurorehabilitation programs.

## INTRODUCTION

Neurological diseases are the largest cause of disability and the second largest cause of death worldwide^1^. Advances in treatment and acute therapies have improved the survival rates of these patients, but many patients continue to experience limitations in their daily activities. As the population and disease processes increase, the demand for rehabilitation services will increase^2^. Neurorehabilitation technologies such as robotic approaches, brain stimulation, virtual reality and assisted walking devices, and various exercise approaches offer innovative and effective approaches^3^. Some important factors increase patient experience and satisfaction in the field of neurorehabilitation. Primarily, the active participation and motivation of patients are critical to the rehabilitation process. Satisfaction also plays an important role in this process^4^. Rehabilitation programs should be tailored to each patient's genetic profile, lesion characteristics, and personal motivation. These programs should be tailored to the experience of the physiotherapist and the specific needs of the patient, which means that the therapist should act as a personal coach, helping patients to set realistic goals and be responsible for their exercise program^5^. However, the integration of technology using computer games, virtual reality, and other interactive tools can increase patients’ engagement and make the rehabilitation process more effective and enjoyable. Consistent evidence for the effectiveness of specific interventions may take time to emerge; however, more intensive and prolonged training than that offered by public health services is required to ensure lasting functional improvements^6^. Consideration of these factors may improve patient satisfaction and outcomes of neurorehabilitation programs by providing an engaging and motivating rehabilitation experience.

The most valid way to evaluate the rehabilitation experience is through a questionnaire survey. Although this process is subjective, all over the world, the only way to assess individual satisfaction is through the assessment of personal opinions. Questionnaires prepared according to scientific content, in which objective questions that accurately reflect the process are selected, are important in understanding the process correctly. In the literature, there are several questionnaires prepared to evaluate the neurorehabilitation experience in this way. Neurorehabilitation Experience Questionnaire (NREQ) is one of the questionnaires prepared in this way^7^. The questionnaires evaluating the neurorehabilitation experience, which are validated and reliable in Turkish, are much more limited. This limitation is one of the important reasons we considered while planning our study. Another one is the introduction of a new questionnaire in Turkish. We aimed to perform the Turkish validity and reliability of the NREQ questionnaire in our study.

## METHODS

### Study design and participants

We conducted our study on neurological patients who received inpatient or outpatient treatment at Akyazı State Hospital between October 2023 and August 2024. Inclusion criteria required participants to be medically stable, capable of understanding the questionnaire, and not have a neurological disease. Patients with dysphasia, cognitive impairment, or comorbid psychiatric disorders were excluded.

Ethical approval for the study was obtained from the Ethics Committee of Sakarya University of Applied Sciences Rectorate with the date 08.09.2023 and number E.96215. The written informed consent was obtained from all participants in accordance with the Helsinki Declaration.

### Procedure

The study was generally planned in two phases. The first phase included the translation and cultural adaptation of the NREQ into Turkish, and the second phase included the reliability and validity analysis of the Turkish NREQ. Before starting the study, permission to use the NREQ was obtained from Ian I. Kneebon, the developer of the scale. During the translation and adaptation phase, we followed the process described by Beaton et al^8^. First, a translation team of four people was formed for the translation of the scale, for which we obtained permission from the responsible author, and the NREQ was independently translated from English into Turkish by three physiotherapists and a neurologist. The translations were compared, and a preliminary Turkish translation of the NREQ was prepared. Subsequently, the translation team back-translated the preliminary Turkish translation from Turkish to English. The contents of the original and translated English versions were compared, differences were noted, and all versions were analyzed to create a synthesis. The translation team discussed and reviewed all materials for Turkish language and cultural adaptation. The activities and words in the questionnaire were not found to be incompatible with the cultural structure of the Turkish society, so no word changes were made. Finally, the final version of the scale was created. A pilot study was conducted with 10 people to assess the comprehensibility of the scale and their data were not included in the study. In the second stage, data collection and analysis processes were carried out for the reliability and validity of the Turkish NREQ.

### Reliability

The reliability and internal consistency of the NREQ were determined by calculating Cronbach's alpha coefficient for the entire scale. The test–retest method was used for the stability of the scale. During this process, the scale was readministered to the same individuals 7 days later. The relationship between the first test and the second test was analyzed using Spearman's correlation analysis. The reliability of the test–retest was determined using the intraclass correlation coefficient.

### Validity

The validity of the NREQ was determined using construct validity and criterion validity. Construct validity was tested by convergent validity and factor analysis methods. Convergent validity was tested using the functional independence scale, which assesses activities of daily living and functional status, and the Perception of Patient Care and Rehabilitation in Older Patients Scale (PCROS), which assesses the rehabilitation service received.

### Outcome measurements

All participants included in the study had their sociodemographic information and health status recorded on the Patient Assessment Form.

### Neurorehabilitation Experience Questionnaire

The NREQ was developed around four main themes: ownership, personal value, holistic approach, and therapeutic atmosphere. These themes were developed based on patient feedback and literature reviews, and a 16-item self-report scale was developed. The pilot questionnaire asked participants to tick either "mostly agree," "not sure," or "mostly disagree" to indicate their agreement with the 16 statements. Participants rated the different therapies they had received, and in the scoring of this question, the responses were summed and divided by the number of agreed items to obtain an average score. The questionnaire consisted of 17 items, increasing by one question in total with the changes made. Response options were "mostly agree"=2, "not sure"=1, and "mostly disagree"=0. The patient experience score ranges from 0 to 36, with higher scores indicating a more positive rehabilitation experience. The total score for the therapy question is calculated by dividing by the number of items indicated and multiplying by two^7^.

### Perception of Patient Care and Rehabilitation in Older Patients Scale

Wressle et al. developed a scale focusing on a patient-centered approach to assess the quality of care and rehabilitation in geriatric patients. There are two subscales of the scale: "Respect and Safety," which reflects how patients feel they are treated by staff, and "Information and Involvement," which assesses patients’ perceptions of being informed and involved in care decisions. The original 5-point Likert scale, which originally consisted of 19 items, was adapted to 17 items due to differences in healthcare practices in Turkey^9^. The total scores ranged from 17 to 85, with higher scores indicating better perceived quality of care^10^.

## RESULTS

The mean age and BMI of the participants were 54.88±14.72 years and 28.55±5.24. The mean number of days of treatment received by the participants was 13.74±7.63. A total of 72 (41.9%) of the participants were female and 100 (58.1%) were male. They received neurological rehabilitation in 136 (79.1%) outpatient and 36 (20.9%) inpatient units. When we look at the neurological patient types of the evaluated patients, 52 people were from Hemiplegia, 40 people were from Parkinson's, 24 people were from Alzheimer's, 18 people were from spinal cord injury, 20 people were from cerebral palsy, and 18 people were from other neurological patient groups.

The average of the first and second evaluation scores of the NREQ questionnaire, which we evaluated for validity and reliability, is given in Table 1. In addition, the total scores of the NREQ and PCROS questionnaires are also given.

**Table 1:** Scores of the Neurorehabilitation Experience Questionnaire and Perception of Patient Care and Rehabilitation in Older Patients Scale questionnaire.

	First evaluation	Second evaluation
	X±SD	X±SD
Item 1	1.88±0.39	1.88±0.32
Item 2	1.56±0.73	1.61±0.65
Item 3	1.63±0.61	1.63±0.57
Item 4	1.93±0.34	1.96±0.26
Item 5	1.79±0.51	1.84±0.48
Item 6	1.90±0.33	1.92±0.27
Item 7	1.95±0.21	1.97±0.18
Item 8	1.97±0.18	1.98±0.13
Item 9	1.93±0.26	1.98±0.15
Item 10	1.84±0.48	1.86±0.41
Item 11	1.84±0.48	1.88±0.44
Item 12	1.94±0.26	1.97±0.17
Item 13	1.88±0.39	1.91±0.29
Item 14	1.95±0.21	1.98±0.15
Item 15	1.95±0.21	1.97±0.17
Item 16	1.70±0.70	1.79±0.55
Item 17	1.91±0.36	1.92±0.27
NREQ 1 total	31.55±2.93	32.04±2.33
PCROS 1 total	79.12±9.42	79.77±7.06

SD: standard deviation; NREQ: Neurorehabilitation Experience Questionnaire; PCROS: Perception of Patient Care and Rehabilitation in Older Patients Scale.

**Figure 1:**
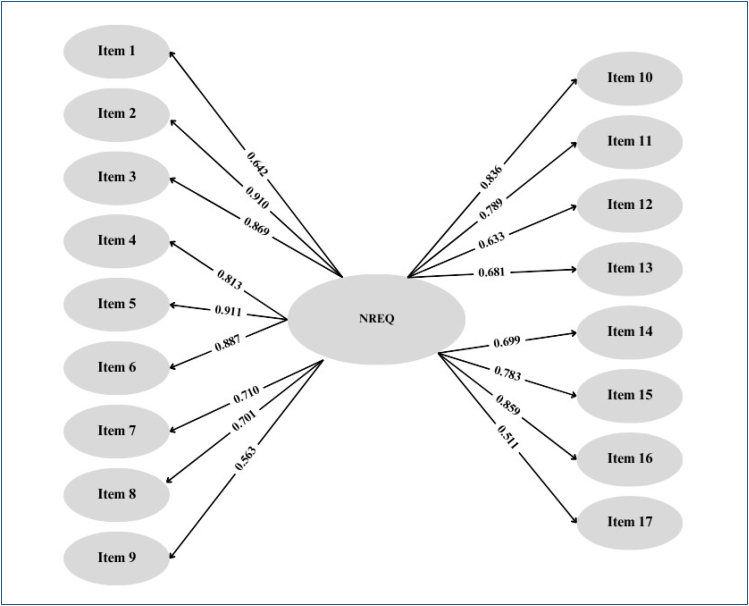
Correlation of Neurorehabilitation Experience Questionnaire items.

The correlation of the first and second evaluations of the NREQ items showed that all items had a high level and positive correlation (see Figure 1).

The correlation between the first and second assessments of the NREQ questionnaire was very high and positively significant (r=0.932, p<0.001). There was a moderate and positive correlation between the first assessment of NREQ and the first assessment of PCROS, and a moderate and positive correlation between the second assessment of PCROS (r=0.440, p<0.001). There was a moderate and positive correlation between the NREQ second assessment and the PCROS first assessment, and a moderate and positive correlation between the PCROS second assessment (r=0.0407, p<0.001).

There is a moderate and significant relationship between the first assessment of NREQ and PCROS total score (R: 0.440, R^2^: 0.194, p<0.001). When the t-test results regarding the significance of the regression coefficients are analyzed, it is seen that there is a significant and positive effect between the first assessment of NREQ and PCROS total score (p<0.001). There is a moderate and significant relationship between the second assessment of NREQ and PCROS total score (R: 0.407, R^2^: 0.166, p<0.001). When the t-test results regarding the significance of the regression coefficients are analyzed, it is seen that there is a significant and positive effect between the second assessment of NREQ and PCROS total score (p<0.001) (see Table 2).

**Table 2:** Regression analysis of the Neurorehabilitation Experience Questionnaire questionnaire and Perception of Patient Care and Rehabilitation in Older Patients Scale questionnaire.

		R	R^2^	F	B	Std. error	Beta	t	p
First scores NREQ and PCROS total	(Constant)				20.735	1.705		12.164	0.000
		0.440	0.194	40.791	0.137	0.021	0.440	6.387	<0.001*
Second scores NREQ and PCROS total	(Constant)				21.291	1.855		11.477	0.000
		0.407	0.166	33.802	0.135	0.023	0.407	5.814	<0.001*

NREQ: Neurorehabilitation Experience Questionnaire; PCROS: Perception of Patient Care and Rehabilitation in Older Patients Scale.

* p<0.05.

## DISCUSSION

The current study aimed to evaluate the validity and reliability of the Turkish adaptation of the NREQ. The results showed that the Turkish version of the NREQ can reliably measure the experiences of patients undergoing neurological rehabilitation. In our study, a significant and highly positive correlation was found between the first and second assessments, supporting the test–retest reliability of the scale. Furthermore, the moderate positive correlation between the NREQ and PCROS contributes to the validity of the Turkish version of the NREQ.

The limited number of questionnaires used to measure neurorehabilitation experience in the literature further increases the importance of this study. In addition, there is no questionnaire measuring neurorehabilitation experience that has been validated in Turkish. The developer of the questionnaire, Kneebone et al., stated that the scale was a reliable and valid questionnaire in the original NREQ study^7^. In the results of the study, internal reliability was confirmed with Cronbach's alpha values of 0.76 and 0.80 at two time points. In addition, test–retest reliability showed a correlation of r=0.70 and concurrent validity was determined by correlations of r=0.32 and r=0.56. The findings of our study are consistent with those of the original study and support the applicability of the NREQ in different cultural contexts. Furthermore, in line with the findings in the literature that new approaches such as tele neurorehabilitation, online rehabilitation assessments, and technology-assisted rehabilitation may improve patient experience, we suggest that it may be useful to evaluate the applicability of the NREQ for such rehabilitation methods.

### Limitations

This study has some limitations. Our sample is only limited, and the generalizability of the findings can be increased with studies to be conducted in a larger population. In addition, further studies in patient groups with different demographic characteristics will contribute to the cultural validity of the scale.

## CONCLUSION

The Turkish adaptation of the NREQ provides a valid and reliable tool for Turkish-speaking health professionals and patients who want to evaluate the neurorehabilitation experience. In this direction, it is thought that the NREQ will be applied on a large population and its use in neurorehabilitation studies in the clinic will increase, contributing to patient satisfaction and obtaining more information about the rehabilitation process.
